# Total Hip Arthroplasty Using a Polished Tapered Cemented Stem in Hereditary Multiple Exostosis

**DOI:** 10.1155/2016/4279060

**Published:** 2016-04-05

**Authors:** Akio Kanda, Kazuo Kaneko, Osamu Obayashi, Atsuhiko Mogami

**Affiliations:** ^1^Department of Orthopaedic Surgery, Juntendo Shizuoka Hospital, Izunagaoka 1129, Izunokuni, Shizuoka 410-2295, Japan; ^2^Department of Orthopaedic Surgery, Juntendo University, Hongo 3-1-3, Bunkyo-ku, Tokyo 113-8431, Japan; ^3^Department of Orthopaedic Surgery, Juntendo Shizuoka Hospital, Nagaoka 1129, Izunokuni, Shizuoka 410-2295, Japan

## Abstract

A 61-year-old Japanese man underwent right total hip arthroplasty for hereditary multiple exostosis. At first presentation, he had suffered from coxalgia for a long time. On radiographic images, there was a gigantic femoral head, increased shaft angle, and large diameter of the femoral neck. He had also developed coxarthrosis and severe pain of the hip joint. The transformation of the proximal femur bone causes difficulty in setting a cementless total hip prosthesis. Therefore, total hip arthroplasty using a cemented polished tapered stem was performed via a direct lateral approach. Using a cemented polished tapered stem allowed us to deal with the femoral bone transformation and bone substance defectiveness due to exostosis and also minimized the invasiveness of the operation.

## 1. Introduction

Hereditary multiple exostosis is a disorder in which an osseous projection capped by cartilage develops on the metaphyses of long bones [1]; the lesion is present in the femoral proximal metaphysis in 25% of patients [2]. Hereditary multiple exostosis of the femoral proximal metaphysis causes the femoral head to become gigantic and the femoral neck diameter to increase and leads to valgus deformity [2, 3]. This results in hip dysplasia and early arthrosis [2]. Changes to the proximal femur cause problems in the setting of a cementless total hip prosthesis. We report a case of hereditary multiple exostosis successfully treated by total hip arthroplasty using a cemented polished tapered stem.

## 2. Case Presentation

**Figure 1:**
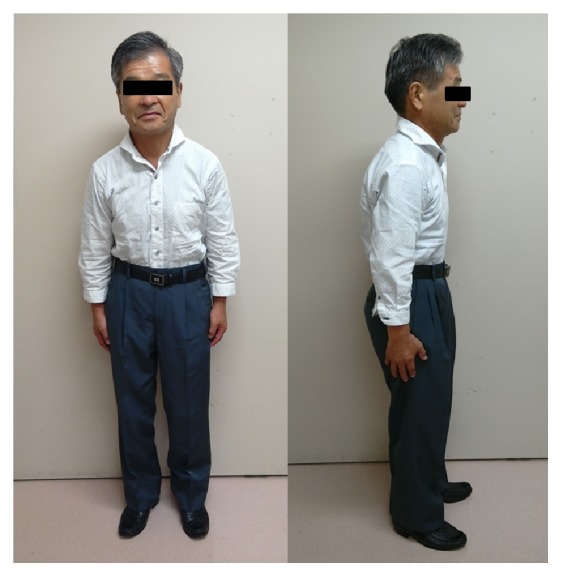
Photographs of the patient in a standing position. He was short, only 149 cm in height.

**Figure 2:**
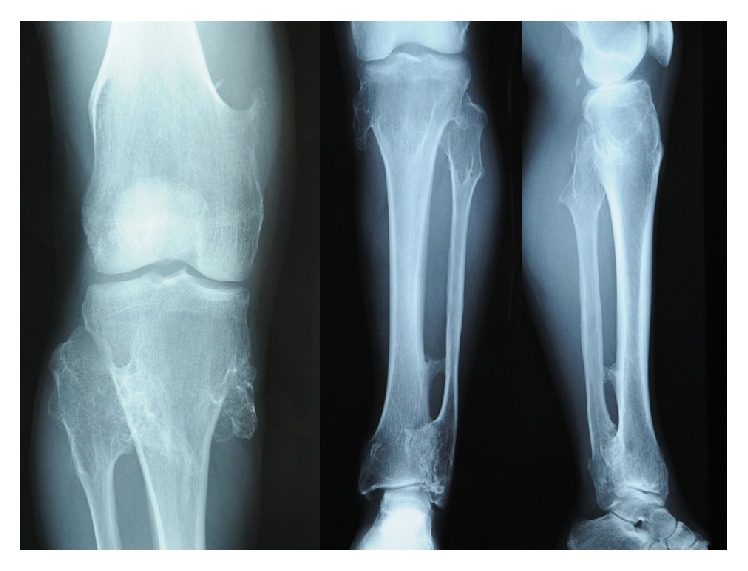
Radiographs of the right knee joint showing osseous projection.

**Figure 3:**
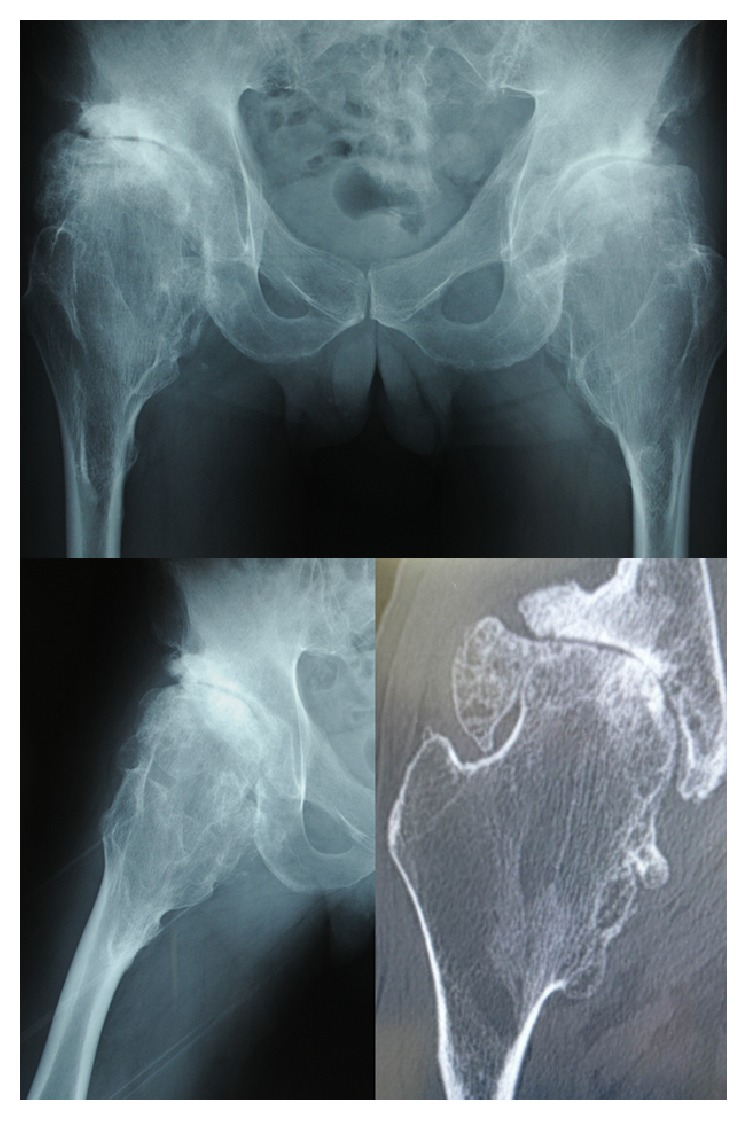
Preoperative radiographs and multidirectional computed tomography image of the right hip joint showing the gigantic femoral head, increasing angle of the neck shaft, and large diameter of the femoral neck.

A 61-year-old Japanese man with coxalgia had a medical examination. His previous history was uneventful; however, he was only 149 cm in height (Figure 1) and had suffered from coxalgia for a long time. Radiography revealed osseous projections at the knee and shoulder joints (Figure 2). On radiographs and multidirectional computed tomography imaging of the hip joint a gigantic femoral head was observed, as well as increased shaft angle and large diameter of the femoral neck (Figure 3). The patient had also developed coxarthrosis and severe pain of the right hip joint. The range of motion of the right hip joint was limited in flexion and abduction. His Japanese Orthopedic Association (JOA) hip joint function score was 45. Radiographs showed stage 4 arthritis based on the JOA hip score, with no ectopic ossification.

**Figure 4:**
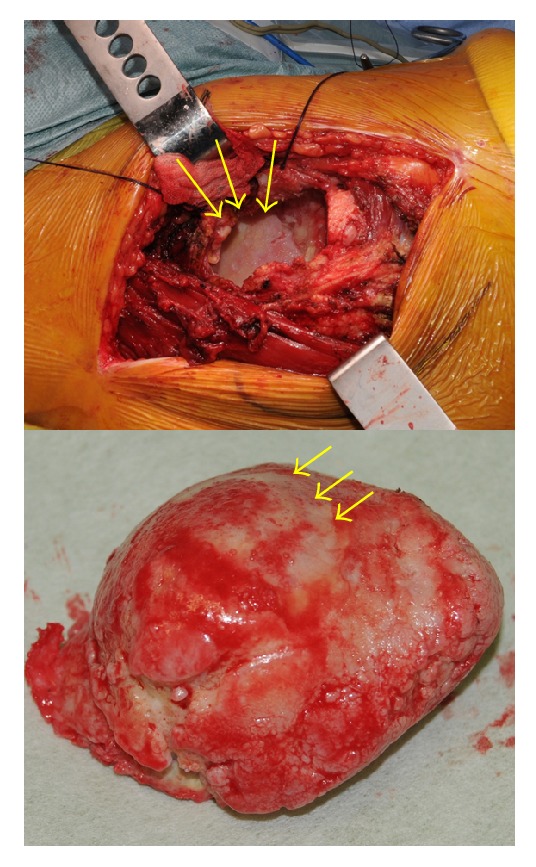
Photograph of the right femoral head in situ and after removal. The acetabular side cartilage and femoral head cartilage showed degeneration (arrows).

**Figure 5:**
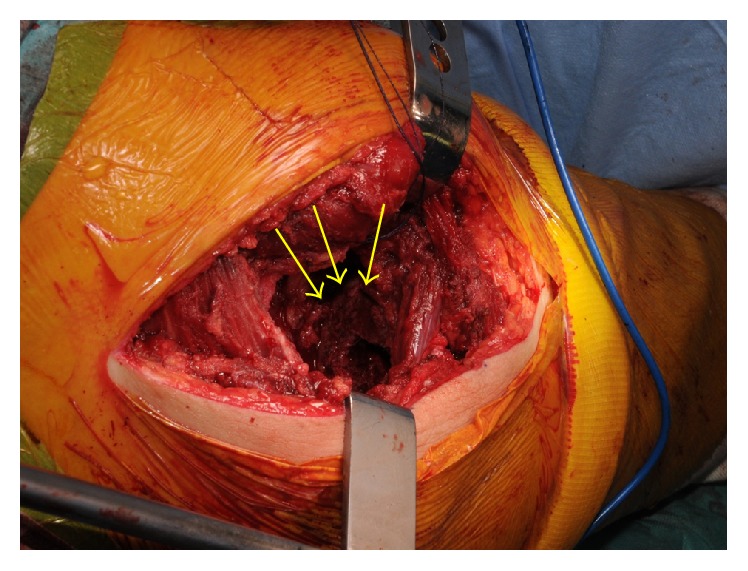
Photograph taken after removal of the right femoral head. The bony substance of the femoral trochanter was poor and the medullary cavity of the proximal femur was expanded (arrows).

**Figure 6:**
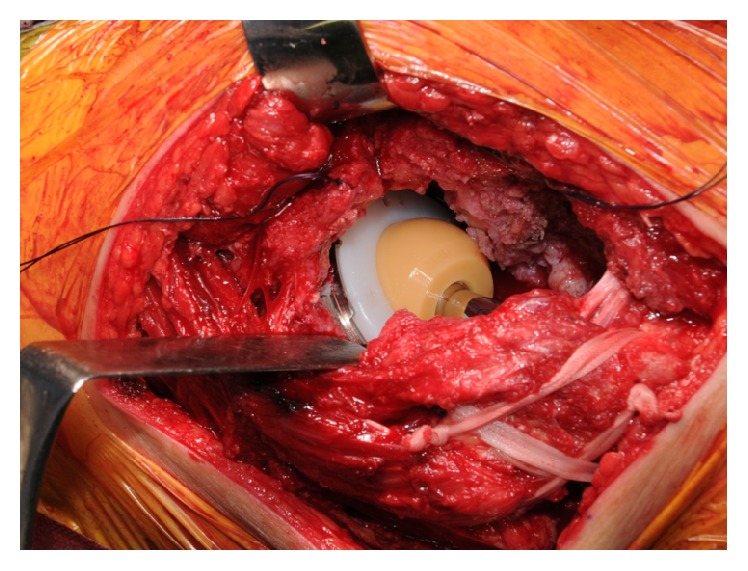
Photograph of the implant in place. We stabilized the fractured greater trochanter with tension band wiring using Kirschner wire and nylon tape.

Right total hip arthroplasty was performed using the direct lateral approach. The soft tissue appeared normal, and an incision was made in the articular capsule. A gigantic femoral head and large diameter of the femoral neck were noted. There was no evidence of exostosis with a cartilage hat. Hip dislocation was difficult because of the gigantic femoral head and caused a trochanteric fracture. The bone substance of the greater trochanter, femoral neck, and femoral trochanter was poor and showed bone fragility. The acetabular side cartilage and femoral head cartilage showed degeneration (Figure 4). We implanted an acetabular cup by press fit without screws at the site of the original acetabulum, because the bone substance there was in good condition. We then performed an osteotomy in a more distal than usual part of the femoral neck, because the bone substance of the femoral trochanter was poor and exostosis was evident in the femoral trochanter on preoperative radiography. We implanted a femoral polished tapered stem using bone cement because of the poor bone substance of the femoral trochanter and the expanded medullary cavity of the proximal femur (Figure 5). We fixed the fractured greater trochanter with tension band wiring using Kirschner wire and nylon tape (Figure 6). We then repaired the articular capsule, gluteus minimus muscle, gluteus medius muscle, and tensor fasciae latae muscle.

**Figure 7:**
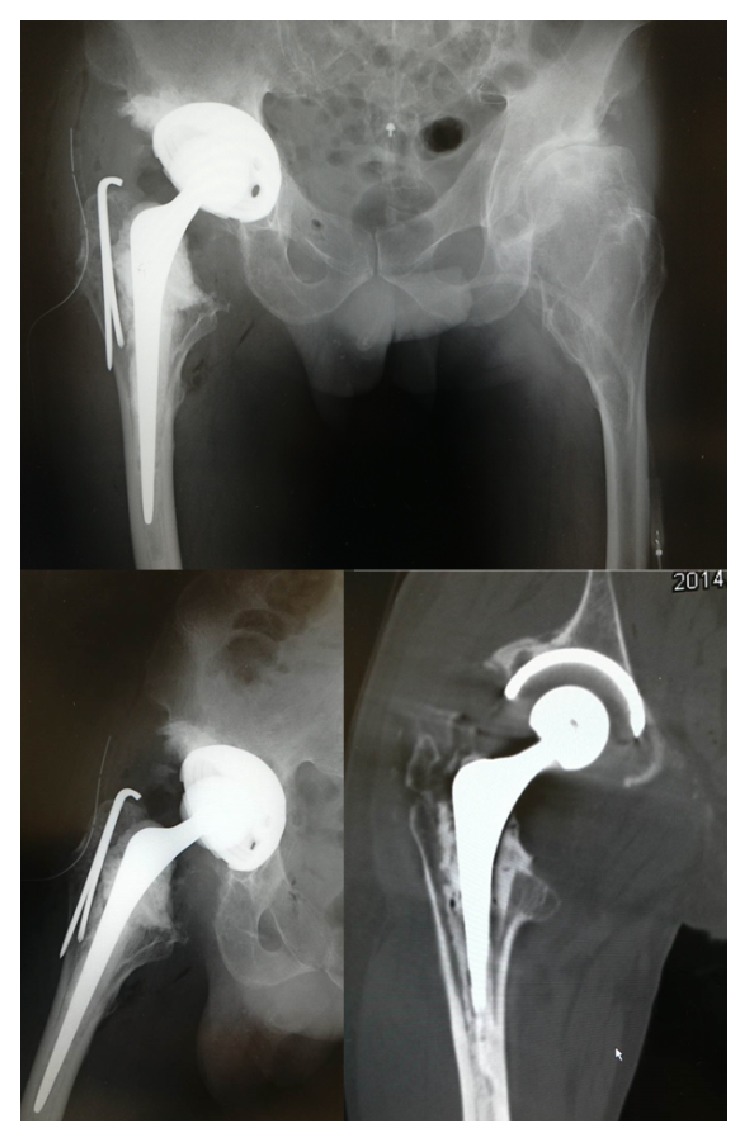
Postoperative radiographs and multidirectional computed tomography imaging showing that the implant setting was unproblematic, and the exostosis at femoral neck had been removed.

On postoperative radiography and multidirectional computed tomography imaging, the implant setting was unproblematic, and the exostosis at the femoral neck had been removed (Figure 7). Postoperatively we permitted walking with full weight-bearing. There were no complications such as dislocation or infection. At postoperative day 29, because we performed rehabilitation over long period so that the range of motion of the right hip joint was limited in flexion and abduction, the patient was discharged from the hospital with a crutch.

**Figure 8:**
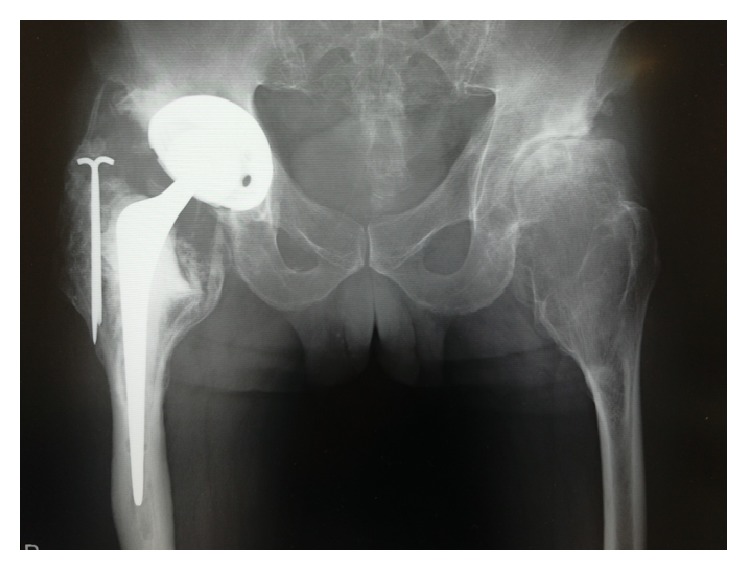
Anteroposterior radiograph of the pelvis taken 13 months postoperatively showing ectopic ossification.

Thirteen months postoperatively the patient had no coxalgia, but the range of motion of the right hip joint was still limited in flexion and abduction. The patient had no limp and no interference with his activities of daily living. His JOA functional test score was 82. There was an ectopic ossification seen radiographically (Figure 8). The patient had returned to his previous work and had no difficulties with everyday activities.

## 3. Discussion

Hereditary multiple exostosis is an autosomal dominant disorder in which an osseous projection capped by cartilage appears on the metaphyses of long bones [1]. The lesion is present in the femoral proximal metaphysis in 25% of patients, resulting in a gigantic femoral head, increased femoral neck diameter, valgus deformity, hip dysplasia, and early arthrosis [2, 3]. The proximal femur also often has the characteristics of fragilitas ossium [4]. This transformation of the proximal femur makes setting a cementless total hip prosthesis difficult. Furthermore, depending on the position of the osseous projection, it may be necessary to perform a more distal femoral osteotomy than usual.

Hereditary multiple exostosis of the femoral proximal metaphysis has previously been treated using very invasive surgical techniques. Moran et al. performed extended trochanteric osteotomy to remove the osseous projection of the proximal femur bone and acetabulum [4]; the proximal cortical bone of the femur was removed to prevent postoperative impingement by the medial projection, and a proximal femoral replacing cementless prosthesis was used to replace the bone defect [4]. Because the extended trochanteric osteotomy allowed the adhesion of the soft tissue to the bone to be maintained, the function of the hip joint function is theoretically maintained [4]. Vaishya et al. also used a cementless prosthesis after osteotomy of the femur to cope with the transformation of the femur [5].

**Figure 9:**
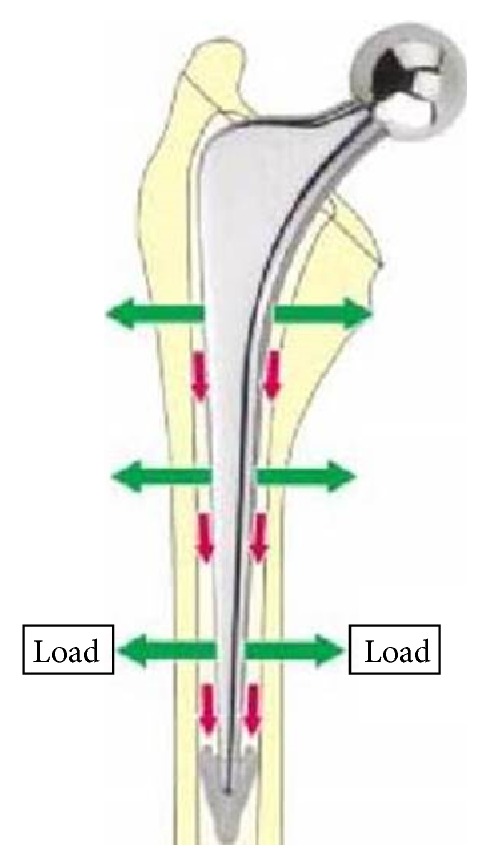
We used a cemented polished tapered stem transmitted load to the laudable distal part of the femur.

To perform less invasive surgery, we used a cemented polished tapered stem. We performed femoral neck osteotomy in the area of the lesser trochanter because of the valgus deformity of the proximal femur, the expansion of the cervical diameter, and the medial osseous projection. Owing to the fragilitas ossium characteristics of the proximal femur, we used a cemented polished tapered stem, which transmitted load to the distal part of the femur (Figure 9). Thirteen months after the operation, heterotopic ossification was present, but there was no local recurrence of the osseous projection, and no complications such as dislocation or infection had occurred.

## 4. Conclusions

This was a case of hereditary multiple exostosis successfully treated by total hip arthroplasty using a polished tapered cemented stem. The femoral bone transformation and bone substance defectiveness due to exostosis can be countered by using a cemented polished tapered stem, which minimizes the invasiveness of the surgery.
